# Skin microbiome differentiates into distinct cutotypes with unique metabolic functions upon exposure to polycyclic aromatic hydrocarbons

**DOI:** 10.1186/s40168-023-01564-4

**Published:** 2023-06-01

**Authors:** Marcus H. Y. Leung, Xinzhao Tong, Zhiyong Shen, Shicong Du, Philippe Bastien, Brice M. R. Appenzeller, Richard J. Betts, Sakina Mezzache, Nasrine Bourokba, Nukhet Cavusoglu, Luc Aguilar, Namita Misra, Cécile Clavaud, Patrick K. H. Lee

**Affiliations:** 1 School of Energy and Environment, City University of Hong Kong, Hong Kong SAR, China; 2 Department of Biological Sciences, School of Science, Xi’an Jiaotong-Liverpool University, Suzhou, China; 3 L’Oréal Research and Innovation, Aulnay-Sous-Bois, France; 4 Human Biomonitoring Research Unit, Luxembourg Institute of Health, Strassen, Luxembourg; 5 L’Oréal Research and Innovation, Pudong, China

## Abstract

**Background:**

The effects of air pollutants, particularly polycyclic aromatic hydrocarbons (PAHs), on the skin microbiome remain poorly understood. Thus, to better understand the interplay between air pollutants, microbiomes, and skin conditions, we applied metagenomics and metabolomics to analyze the effects of PAHs in air pollution on the skin microbiomes of over 120 subjects residing in two cities in China with different levels of air pollution.

**Results:**

The skin microbiomes differentiated into two cutotypes (termed 1 and 2) with distinct taxonomic, functional, resistome, and metabolite compositions as well as skin phenotypes that transcended geography and host factors. High PAH exposure was linked to dry skin and cutotype 2, which was enriched with species with potential biodegradation functions and had reduced correlation network structure integrity. The positive correlations identified between dominant taxa, key functional genes, and metabolites in the arginine biosynthesis pathway in cutotype 1 suggest that arginine from bacteria contributes to the synthesis of filaggrin-derived natural moisturizing factors (NMFs), which provide hydration for the skin, and could explain the normal skin phenotype observed. In contrast, no correlation with the arginine biosynthesis pathway was observed in cutotype 2, which indicates the limited hydration functions of NMFs and explains the observed dry skin phenotype. In addition to dryness, skin associated with cutotype 2 appeared prone to other adverse conditions such as inflammation.

**Conclusions:**

This study revealed the roles of PAHs in driving skin microbiome differentiation into cutotypes that vary extensively in taxonomy and metabolic functions and may subsequently lead to variations in skin–microbe interactions that affect host skin health. An improved understanding of the roles of microbiomes on skin exposed to air pollutants can aid the development of strategies that harness microbes to prevent undesirable skin conditions.

Video Abstract

**Supplementary Information:**

The online version contains supplementary material available at 10.1186/s40168-023-01564-4.

## Introduction

The human skin hosts a diverse community of microbes (i.e., microbiome) on the stratum corneum layer of the epidermis. The microbiome is indispensable for the modulation of host immunity, protection against opportunistic pathogens, and maintenance of skin physiology [1]. More importantly, skin conditions such as acne [2, 3] and atopic dermatitis [4, 5] have microbial contributions as part of their etiology, and skin microbiome dysbiosis may partly explain the onset of these conditions. Given the interconnectedness between the skin microbiome and host skin health, examining how host physiological and environmental factors shape the skin microbiome may allow the metabolic functions of microbes to be harnessed to protect hosts against various skin conditions. The understanding of the skin microbiome has increased in recent years owing to metagenomics sequencing [6–8] and mass-spectrometry-based profiling of microbial metabolites [9–11].

Studies have identified distinguishable microbial sub-community clusters termed cutotypes [12] (or cutaneotypes [13]) in host skin, which differ in microbial diversity, composition, functional, and antimicrobial resistance profiles. The distribution of cutotypes may vary with skin characteristics of individual hosts and thus help explain how alterations in skin microbial taxonomic composition can lead to the onset of skin conditions across different demographic groups. However, little information is currently available on factors that explain the differentiation of cutotypes and how this relates to skin health. Such knowledge would undoubtedly aid the identification of cutotype markers associated with undesirable skin conditions, thereby assisting the development of personalized solutions for improving skin health.

Chronic exposure to air pollutants is a serious concern for individuals living in urban environments. Many pollutants occur in the atmosphere, of which polycyclic aromatic hydrocarbons (PAHs) are among the most hazardous class of organic molecules to human health [14]. Earlier reports on the impacts of air pollution on skin have pointed toward an association with premature skin aging [15–18]. Specifically, cutaneous exposure to PAHs has been linked to changes in the physiological properties of the skin, potentially leading to pigmented spots [11] and cancer [19]. While specific members of the skin microbiome have been shown to metabolize PAHs [20, 21], the roles of PAH exposure in shaping the skin microbiome warrant further investigation. In our previous study [22], we demonstrated a dose–response relationship between the levels of exposure to various PAHs and changes in the taxonomic compositions of skin microbiomes, as well as variations in the abundances of microbial functional genes crucial for host–microbe interactions, virulence, and host immune modulation. However, these findings were largely based on amplicon sequencing of the phylogenetic marker genes of bacteria and fungi, while functional profiling by metagenomics sequencing was performed for only a small subset of samples with no corresponding metabolite characterization. More importantly, studies have yet to answer whether and how PAH exposure drives skin microbiome differentiation, what is the relationship between PAH exposure and cutotype differentiation, and whether any changes in cutotypes associated with PAH exposure can also result in changes in the function and metabolites of the microbiome as well as the skin–microbe interactions that may be detrimental to host skin health.

To address these questions, we performed a large-scale study of the facial cheek microbiomes of 124 Chinese females from two cities with different PAH exposure levels. The concurrent assessment of the metagenomes and metabolites allowed comprehensive characterization of the dynamic interplays between PAH exposure, skin microbiomes, and their functional potentials and metabolites. We report, for the first time, a link between PAH exposure from air pollution and the differentiation of the skin microbiome into cutotypes with distinct taxonomic, functional, resistome (i.e., the repertoire of antibiotic resistance genes), and metabolite compositions as well as skin phenotypes.

## Results

### Differentiation of skin microbiomes into distinct cutotypes was associated with PAH exposure

We detected 1525 species-level taxa at an average relative abundance of > 0.00001% of all the skin microbiomes, with 42 of these taxa considered core (i.e., present across all samples) as they comprised an average of nearly 80% of the reads per sample (Supplementary Fig. 1a). The microbial diversity was higher in subjects from the more polluted city of Baoding (Supplementary Fig. 1b). Fungal reads composed an average of 2.2% (95% confidence interval 0–8.7%) of all the skin microbiomes, most of which were classified as *Malassezia restricta* and *Malassezia globosa* (Supplementary Fig. 1c-d). Viral and archaeal reads cumulatively composed an average of 0.05% (95% confidence interval 0–0.36%) of the microbiome across each sample.

**Fig. 1:**
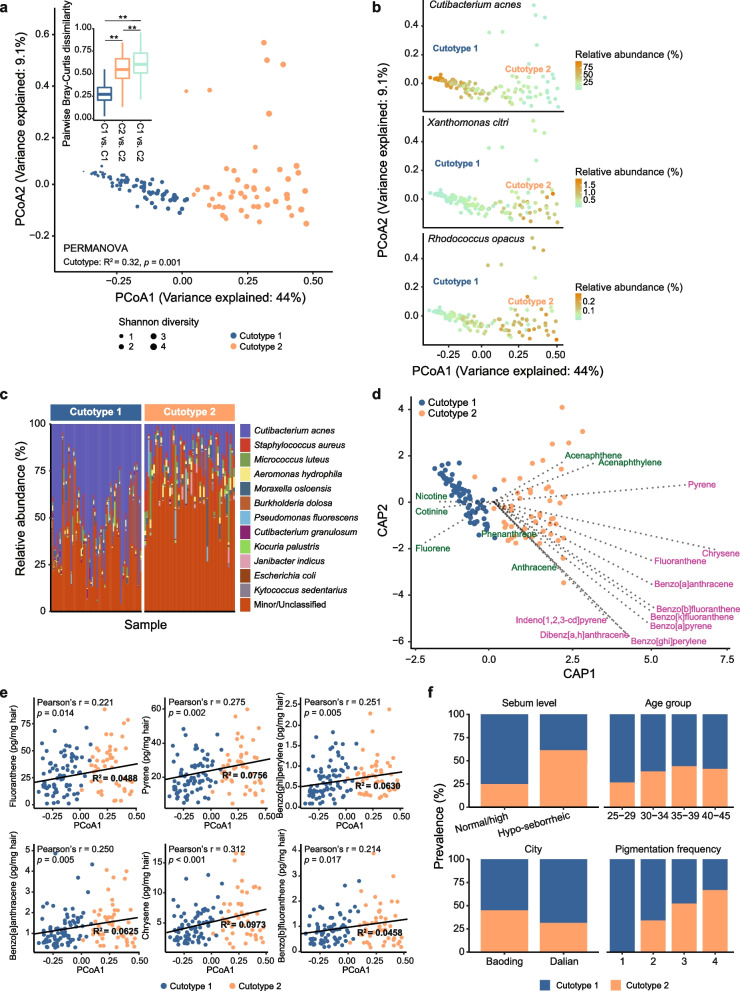
Cheek microbiomes were differentiated into cutotypes and associated with PAH exposure. **a** Principal coordinate analysis (PCoA) plot based on the Bray–Curtis dissimilarity of microbial community composition. Data points represent metagenomic samples with point size representing Shannon diversity and color showing the cutotype as determined by the prediction strength analysis. The inset panel shows the pairwise Bray–Curtis dissimilarity between samples of the same or different cutotypes, showing that microbiomes within a cutotype were more similar than between cutotypes (**Tukey’s pairwise post-hoc *p* < 0.05 for all pairwise comparisons). **b** PCoA plot as shown in panel **a** with the data points colored according to the relative abundance of *Cutibacterium acnes*, *Xanthomonas citri*, and *Rhodococcus opacus* in cutotypes 1 and 2. **c** Top 12 species-level bacterial taxa based on the average relative abundance in all the samples organized by cutotype. Other bacterial taxa were grouped into the “Minor/Unclassified” category. **d** Distance-based redundancy analysis depicting the effects of exposure to high and low molecular weight PAHs (purple and green, respectively) on cutotype differentiation. PAHs with a molecular weight < 200 g/mol were considered low. **e** Spearman’s correlation between the first component of the PCoA and the measured concentrations of the six PAHs. Linear regression lines and *R*^*2*^ values are shown. **f** Association of samples in the respective cutotypes with different host factors and host skin phenotype

Prediction strength analysis estimated the presence of two distinct clusters among the samples. These two distinct community clusters, which explained 32.3% of the total community variation (Supplementary Table 1), were also detected with the Bray–Curtis dissimilarity (Fig. 1a). The microbiome of one cluster (hereafter, cutotype 1), which had a relatively lower Shannon diversity, was enriched with *Cutibacterium acnes* and its taxonomic relative *Cutibacterium granulosum* (Fig. 1a–c). Alternatively, the second cluster (hereafter, cutotype 2) had a relatively higher microbial diversity with > 100 species-level taxa (e.g., *Xanthomonas citri* and *Rhodococcus opacus*), including common skin colonizers such as *Staphylococcus aureus* and *Corynebacterium* species (Fig. 1a–c).

Cutotype 2 was enriched with a diverse array of genera (e.g., *Paracoccus*, *Archromobacter*, *Caulobacter*, *Pseudomonas*, *Rhodococcus*, and *Sphingomonas*) known to have biodegradation capabilities (Supplementary Table 2) and some of them were found in PAH-degrading microbial communities [23, 24]. We thus hypothesized that the differentiation of the microbiome into cutotype 2 was associated with PAH exposure. Indeed, distance-based redundancy analysis revealed that exposure to many different PAHs appeared to drive the microbiome toward cutotype 2 (Fig. 1d). Exposure to a small number of lower-molecular-weight PAHs (i.e., < 200 g/mol) was associated with the differentiation of all cutotype 1 microbiomes and a small subset of cutotype 2 microbiomes, whereas exposure to a large number of higher-molecular-weight PAHs (i.e., > 200 g/mol) was associated with the differentiation of most cutotype 2 microbiomes. The exposure levels to many PAHs were also correlated with the first dimension of the community compositional principal coordinate axis (i.e., PCoA1 in Fig. 1a) (Fig. 1e), suggesting that PAH exposure and community composition are partially linked.

Cutotype was not related to age group and only marginally associated with city (Fig. 1f, Supplementary Fig. 1e and Supplementary Table 1). Subjects with dry skin (sebum level < 70 μg/cm^2^) were prone to having a microbiome resembling cutotype 2, while those with normal to high sebum levels (> 70 µg/cm^2^) were prone to having a microbiome resembling cutotype 1 (Fig. 1f, chi-square with Yates correction = 14.4, *p* = 0.00015), which was consistent with the high abundance of *Cutibacterium*. Although not statistically significant, subjects with higher pigmentation frequency were more prone to having a microbiome resembling cutotype 2 (Fig. 1f). Regarding fungal taxa, community differences could neither be explained by cutotypes nor other host factors, and shifts along PCoA1 based on the Bray–Curtis dissimilarity were only associated with exposure to nicotine (Spearman’s *ρ* = 0.308, *p* = 0.018) and cotinine (Spearman’s *ρ* = 0.251, *p* = 0.045).

### Species-specific correlations between growth rate and PAH exposure

**Fig. 2:**
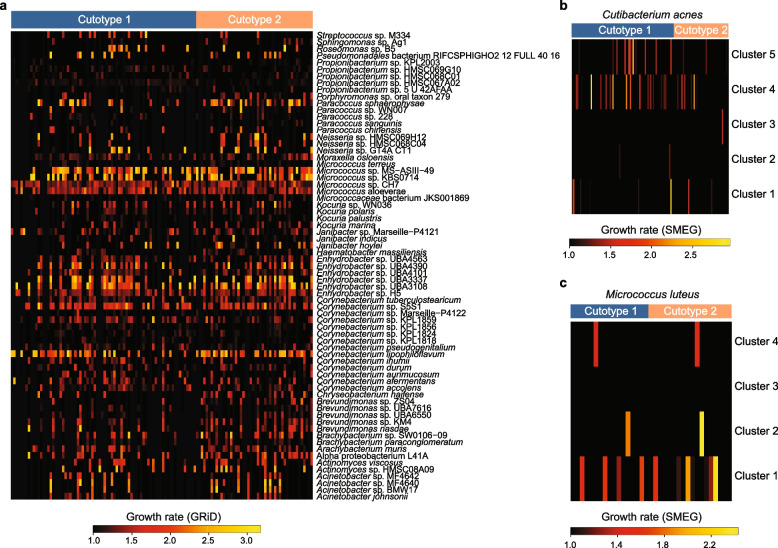
Growth rate inference of the cheek microbiome at the species and subspecies levels. **a** Heatmap of the species level inferred growth rates for taxa across samples grouped by cutotype. **b**, **c** Heatmaps of the inferred growth rates for subspecies taxa belonging to **b** *Cutibacterium acnes* and **c** *Micrococcus luteus*

Growth rate inference [6, 7] was performed to assess how cutotypes and PAH exposure may influence microbial physiology. Our findings suggested that taxa in the genera of *Micrococcus* and *Corynebacterium* were among those with the highest inferred growth rate and it was similar between the two cutotypes (Fig. 2a). Taxa within *Enhydrobacter*, a genus phylogenetically related to *Moraxella* and hypothesized to be enriched on Asian skin [12, 25, 26], were also inferred to have a high growth rate. The levels of exposure to dibenzo[a,h]anthracene and acenaphthene were associated with changes in the inferred growth rates of two bacterial species (i.e., a bacterium affiliated with *Micrococcaceae* and *Janibacter indicus*) (Supplementary Table 2). City, acne onset, and pigmentation frequency were not associated with changes in the inferred growth rates of any bacterial taxa. Similarly, single-nucleotide polymorphism-based subspecies-level growth rate estimation [9] did not indicate cutotype-based growth pattern differences between the clusters of related taxa within *C. acnes* (Fig. 2b) and *Micrococcus luteus* (Fig. 2c). Inferred growth rates of *C. acnes* and *M. luteus* subspecies taxa on the skin were comparable to those reported in previous studies [6, 7, 27]. Overall, pollution exposure did not appear to influence inferred growth rates across the microbiome.

### PAH-associated cutotypes were enriched in diverse potential functions

**Fig. 3:**
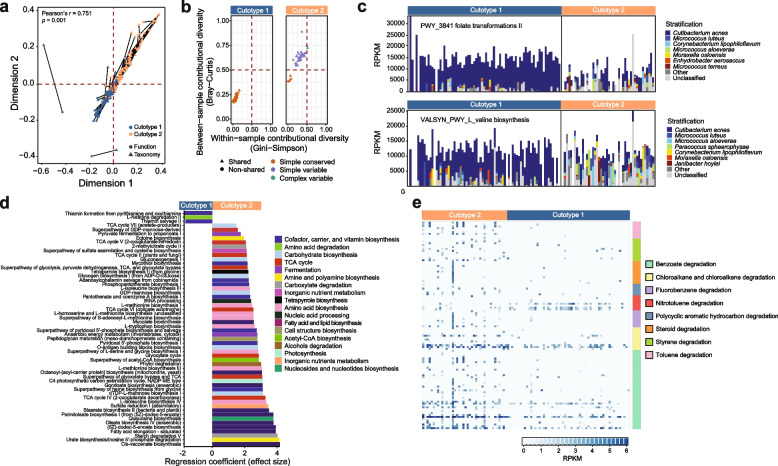
Cutotypes differed in contributional functional diversity and enriched functional pathways. **a** Procrustes analysis showing congruence between the taxonomic and functional compositions of the microbiome. The statistically significant Pearson correlation indicates that microbiomes were similar in both taxonomic composition and functions. **b** Contribution diversity differences between cutotypes of core gene UniRef90 pathways (those present in ≥ 75% of the samples within each cutotype). Core pathways detected in both cutotypes are considered to be “shared” (triangles). **c** Examples of core pathways (folate transformation II and L-valine biosynthesis) show differences in contributional diversity between cutotypes. Relative abundance is expressed in reads per kilobase per million reads (RPKM). **d** Enrichment of UniRef90 pathways between cutotypes according to MaAslin2. Only statistically significant (adjusted *p* < 0.05) pathways are shown. **e** Relative abundance in RPKM of KOs involved in xenobiotic degradation across samples in the respective cutotypes

The functional profile was examined to understand how compositional differences between cutotypes translated to variations in functional potentials. The congruence between taxonomy and functional pathways in the skin microbiomes in cutotypes 1 and 2 (Fig. 3a) suggested that samples with similar taxonomic compositions also tended to have similar functions. As in taxonomy, cutotype was the strongest factor in explaining functional differences between microbiomes, as compared to other parameters such as clinical or city factors (Supplementary Table 1).

The core set of UniRef90 pathways (i.e., ≥ 75% of samples in each cutotype) was assessed by contributional diversity analysis to further compare the pathway diversity between cutotypes. For both cutotypes, the core was dominated by pathways involved in the biosynthesis and metabolism of nucleotides and amino acids (Supplementary Table 3). Despite this conservation, core pathways for cutotype 1 mainly exhibited low within- and between-sample diversity (i.e., “simple and conserved” contributional diversity [28]) (Fig. 3b). In contrast, core pathways in cutotype 2 were dominated by those with high between sample diversity (i.e., “simple and variable” and “complex and variable” contributional diversity [28]) (Fig. 3b). The discrepancy between the two cutotypes regarding within and between sample variations was most likely due to the dominant contribution of *C. acnes* in cutotype 1 to the shared core pathways (e.g., folate transformation and valine biosynthesis) (Fig. 3c).

Multivariate analysis was performed to detect the enrichment of KEGG Orthology (KO) gene families between cutotypes, which revealed the enrichment of 556 KOs (Supplementary Table 2) and 53 UniRef pathways spanning multiple facets of microbial physiology (Fig. 3d) in cutotype 2 versus cutotype 1. At the pathway level, cutotype 1 was characterized by amino acid degradation potential (PWY_5028: L-histidine degradation III), while cutotype 2 was associated with multiple amino acid biosynthesis pathways (*n* = 8), including tryptophane (TRPSYN_PWY: L-tryptophan biosynthesis) and methionine biosynthesis (*n* = 4). Cutotype 2 was also characterized by an increased number of pathways related to carbohydrate biosynthesis (*n* = 6) and the tricarboxylic acid (TCA) cycle (*n* = 7), suggesting a shift in the microbial metabolism.

Given that high PAH exposure was linked to microbiome differentiation into cutotype 2 concomitant with the enrichment of pathways related to diverse functions, we hypothesized that this cutotype presented an increased ability to degrade xenobiotic compounds. To test this hypothesis, KOs associated with xenobiotic compound degradation were compared between the two cutotypes. The Mann–Whitney test results showed an increased abundance of KOs involved in the degradation of benzoate (FDR-adjusted *p* = 6.6 × 10^–16^), chloroalkane/alkene (FDR-adjusted *p* = 7.2 × 10^–16^), PAH (FDR-adjusted *p* = 6.9 × 10^–5^), steroids (FDR-adjusted *p* = 9.9 × 10^–5^), styrene (FDR-adjusted *p* < 7.8 × 10^–10^), and toluene (FDR-adjusted *p* < 5.7 × 10^–14^) in cutotype 2 (Fig. 3e). Conversely, abundances of KOs linked to nitrotoluene degradation were increased in cutotype 1 (FDR-adjusted *p* = 3.78 × 10^–5^). Overall, these results suggested that while each cutotype was characterized by distinct microbes that contributed to shared core functions, they also presented differential enrichment of metabolic functions that conferred adaptive and biodegradative advantages given certain environmental conditions.

Cutotypes also had different resistomes, which may explain cutotype-specific responses to host antimicrobial treatment. The most abundant gene family markers were those conferring resistance to fluoroquinolones, aminoglycosides, and beta-lactams (Supplementary Table 4). Cutotype explained nearly 18% of resistome variation (Supplementary Table 1), with point mutations in *gyrA* (ARO: 3,003,974) conferring resistance to fluoroquinolone and an efflux protein (tet(V)) conferring resistance to tetracycline (ARO: 3,000,181) enriched in cutotype 2. Exposure to PAHs also positively correlated with the abundance of a small number of antibiotic resistance gene families (Supplementary Table 2), including those encoding for a tetracycline efflux pump (tet(33)) and several plasmid- or integron-encoded gene families related to aminoglycoside resistance. Thus, these results suggest that chronic exposure to PAH may favor the presence of species with antimicrobial resistance genes.

### Skin metabolites were specific to cutotypes and correlated with the microbiome and PAHs

In our earlier report using 16S rRNA gene amplicon sequencing data [11], we showed that the skin metabolome accounted for one third of the variability in bacterial diversity. Here, we further investigated the links between metabolome and metagenomic data. The metabolomic profiles of subjects [11] were examined with the corresponding metagenomes to test whether the molecules on skin were specific to cutotypes. Many gamma-glutamyl amino acids, xenobiotics, and unknown metabolites were overrepresented in cutotype 2 (Supplementary Fig. 2). In contrast, many diverse lipid molecules, largely represented by the sebum components myristic and palmitoleic acid [29], were overrepresented in cutotype 1.

Correlation network analysis was performed by integrating metabolomic data with corresponding taxonomic, functional, metabolomic, and PAH features for each cutotype to (i) provide a comprehensive and multifaceted view of the potential ecological relationships between pollutant exposure and microbial and metabolic features and (ii) compare the relationships between microbiome components of the two cutotypes. While networks of the cutotypes presented similar numbers of features (i.e., nodes), the network of cutotype 1 was more integrated with a higher number of edges and average number of neighbors per node (Supplementary Table 5). The network density of cutotype 1 also exceeded that of cutotype 2, which suggested a more stable network in the former.

**Fig. 4:**
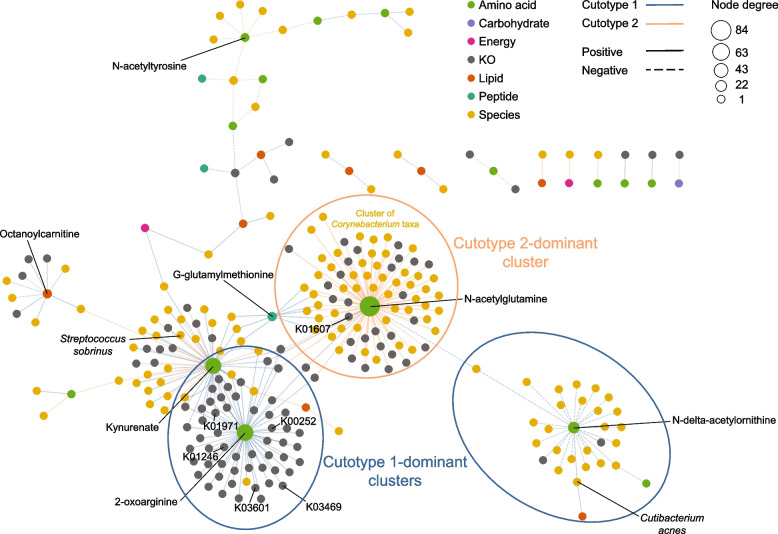
Cutotype-specific correlations between compositional, functional, metabolomic features, and PAH exposure. Each node represents a feature and is colored by feature type and sized by node degree. Blue and orange edges represent those in cutotypes 1 and 2, respectively. Solid and dashed edges represent positive and negative correlations, respectively, with line thickness representing the absolute value of the Spearman’s correlation. Only metabolites considered enriched in the polluted city of Baoding [11] are shown to enhance visual clarity. Nodes connected to *N*-acetylglutamine are circled to highlight a cluster of *Corynebacterium* taxa. The species-level taxonomy of these *Corynebacterium* nodes, as well as all other significant correlations, can be found in Supplementary Table 5. The multivariate analysis was performed using HallA. Correlations with corrected *q* value ≤ 0.25 are statistically significant [30], and only those with a Spearman’s correlation >|0.25| were included in analysis

To observe the overall correlations between the microbiome features of the two cutotypes, their networks were merged, which produced 2684 nodes and 52,727 correlations (Supplementary Table 5). Significant correlations involving metabolites previously [11] shown to be associated with undesirable skin phenotypes (e.g., N-acetyl and G-glutamyl amino acids, urea cycle intermediates, amino acids of tryptophan metabolism, fatty acids and carnitine lipids, lactate, and TCA cycle metabolites) were observed (Fig. 4). N-acetylglutamine, a metabolite of arginine biosynthesis, was central to the network and had significant positive correlations with multiple *Corynebacterium* species (*n* = 43) and KOs (*n* = 30) (e.g., K01607 that encodes 4-carboxymuconolactone decarboxylase, an enzyme involved in benzoate degradation) in cutotype 2 (Supplementary Table 5 and Fig. 4). N-acetylglutamine was also correlated with the *fpr* gene (K00528), which encodes an oxidoreductase, as well as KOs of the TCA cycle, carbohydrate metabolism, and biofilm formation (Supplementary Table 5). These functions are representative of the bacterial degradation strategy for aromatic compounds, in which compounds are de-aromatized and then funneled into central carbon pathways such as the TCA cycle [23]. The strong positive correlations with *Corynebacterium* species suggested that the cluster may contribute to PAH biodegradation. Other species capable of biodegradation, including *Pseudomonas* and *Rhodococcus*, were also positively correlated with N-acetylglutamine (Supplementary Table 5). For cutotype 1, two clusters were observed. One cluster featured species, including *C. acnes*, and KOs that were positively correlated with N-delta-acetylornithine, which is another arginine pathway metabolite. The second cluster showed positive correlations with 2-oxoarginine and many KOs (*n* = 56) encoding base and mismatch repair and related functions (K03469, K01246, K03601, and K01971). 2-Oxoarginine was also positively correlated with glutaryl-CoA dehydrogenase (K00252) for benzoate degradation and glutamate dehydrogenase (K00262) for arginine biosynthesis (Fig. 4).

Kynurenate, a tryptophan pathway metabolite, which may stimulate the onset of inflammatory responses in hosts [31], showed positive correlations with the oral bacterium *Streptococcus sobrinus* in cutotype 2. Interestingly, the presence of oral bacteria on the skin has been attributed to skin aging [32]. An unknown metabolite, X-13737, previously shown to be enriched in subjects from the more polluted city of Baoding [11], showed a positive correlation in cutotype 2 with a taxon affiliated with *Pseudomonas*, which has been postulated to compete with resident skin *Cutibacterium* [33].

## Discussion

The skin microbiome is shaped by the external environment surrounding the skin [34], and subsequently, exposure to pollutants such as PAHs has been linked to a wide range of skin conditions [14, 19, 35, 36]. Recently, the roles of the skin microbiome in modulating host responses to environmental stressors have gained recognition [37]. By analyzing 32 metagenomic samples from cheeks and scalps, we previously associated PAH exposure with changes in the compositional and functional capabilities of the skin microbiome, with potential consequences for skin phenotype [22]. In this study, by analyzing the metagenomes and corresponding metabolites from the cheeks of over 120 subjects, we provided evidence that PAH exposure could play a role in the differentiation of skin microbiomes into cutotypes that have distinct compositions and metabolic functions at the community and genomic levels and are associated with different skin phenotypes. The link between PAH exposure and cutotype differences may help explain the potential roles of the microbiome in modulating host skin health upon air pollutant exposure.

The presence of cutotypes on the skin of subjects in this study was consistent with the stratification of microbiomes on other human body parts, which has been linked to changes in host phenotypes, physiology, and susceptibility to diseases [38–40]. A recent study of nearly 300 Chinese individuals also reported that skin microbiomes can be grouped into two cutotypes [12], with the differentiation mainly driven by nutrient availability as well as host physiology and chronological age [8]. Our study showed that cutotype differentiation may also occur as a function of environmental exposure to air pollution. The bacterial taxa previously documented to have biodegradation capabilities (e.g., *Archromobacter*, *Caulobacter*, *Paracoccus*, *Pseudomonas*, *Rhodococcus*, and *Sphingomonas*) [23, 24] were enriched in cutotype 2, and a positive correlation was detected between acenaphthene exposure level and the inferred growth rate of a species in *Janibacter*, a genus capable of using multiple PAH types as carbon sources [41]. These observations together with the documented PAH metabolic capabilities of common skin colonizers [20, 21] suggest that PAHs are actively metabolized by the skin microbiome. The species with biodegradation abilities may have been originated from the environment [24, 42] and managed to transiently colonize on skin due to the presence of PAHs. However, PAHs do not appear to greatly influence fungal community composition. The cutotypes also showed significant resistome differences, which was consistent with previous cutotype analyses [12]. The roles of PAH exposure in driving resistome variations via cutotype differentiation remain to be elucidated.

Multivariate analysis of metagenomics, metabolomics, and PAH exposure data revealed complex associations between pollutant exposure and various aspects of the skin microbiome. The correlation network of cutotype 2 was strongly associated with exposure to multiple PAHs and had a reduced integrity, which has been previously associated with acne and dandruff on the cheeks and scalp [22, 43]. However, whether microbiome network stability can be used as a diagnostic marker to test for susceptibility to pollutant-related skin conditions requires further investigation. Each PAH appeared to have unique and cutotype-specific associations with microbial features. Metabolites previously detected at higher levels in the more polluted city [11] showed cutotype-specific associations with functional genes involved in DNA repair, secretion systems, and pollutant degradation. While we cannot determine whether the detected metabolites on the skin originated from microbial metabolism, environmental exposure, or anthropogenic factors [10], the presence of shared but opposing correlations between cutotypes suggest that specific microbes interact differently with chemicals on the skin.

**Fig. 5:**
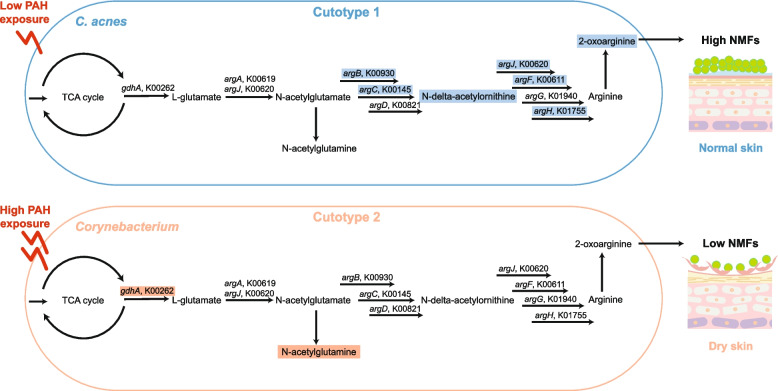
Schematic illustration of the potential influence of the arginine biosynthesis pathway in cutotypes 1 and 2 on natural moisturizing factors (NMFs) for skin hydration. Arginine biosynthesis played an important role in cutotype 1, but only a limited role in cutotype 2. The illustration was drawn based on the key correlations found between the dominant taxa, metabolic functions, and metabolites in the respective cutotypes. The KOs and metabolites highlighted by a rectangular box are those identified as having significant positive correlations in the network analysis

The two identified cutotypes were associated with distinct skin dryness and microbial metabolism in which the arginine pathway appeared to play a pivotal role (Fig. 5). Cutotype 1, which was mostly associated with normal to greasy skin, was dominated by *C. acnes* (consistent with previous reports on sebaceous sites [8]) and functions related to lipid metabolism and amino acid biosynthesis. *C. acne*s, among other taxa, was positively correlated with N-delta-acetylornithine as well as many KOs (i.e., K00619 [*argA*], K00930 [*argB*], K00145 [*argC*], K00611 [*argF*], K01755 [*argH*], and K00620 [*argJ*]) in the arginine biosynthesis pathway, suggesting that arginine is produced in cutotype 1. Arginine is a key amino acid in filaggrin-derived natural moisturizing factors (NMFs), which provide hydration for the skin [44]. We thus speculate that arginine from bacteria contributes to skin hydration (Fig. 5). High-throughput mass spectrometry proteomic analysis of the cheek stratum corneum of a subset of subjects (34 from cutotype 1 and 19 from cutotype 2) revealed that the expression level of the FLG gene-encoded filaggrin [45] was similar between the two cutotypes (data not shown), suggesting that skin dryness does not appear to be linked to the FLG gene.

In contrast, cutotype 2 was associated with dry skin and hyperpigmentation in subjects aged under 45. Diverse species, including *Paracoccus* [46], *Sphingomonas* [23], and *Pseudomonas* [20], that can reportedly degrade PAHs were found in cutotype 2. In particular, members of *Corynebacterium* were the key taxa in cutotype 2. These taxa were positively correlated with benzoate degradation and KOs in the TCA cycle (i.e., K01607 and K00262 [*gdhA*]). Growth in the presence of aromatic compounds has been shown to upregulate enzymes in the TCA cycle of *Corynebacterium glutamicum* (e.g., a fivefold increase in GdhA that leads to L-glutamate production) [47], which we speculate is similar to that in the microbiome of skin exposed to PAHs. Consistent with the potential increase in L-glutamate, the metabolite N-acetylglutamine, produced from L-glutamate, was positively correlated with *Corynebacterium* taxa (Fig. 5). In contrast to cutotype 1, *Corynebacterium* taxa in cutotype 2 were not correlated with any KOs or metabolites in the arginine biosynthesis pathway, and positive correlations with KOs in the arginine biosynthesis pathway were only found in taxa with low relative abundances. Thus, arginine synthesis by the microbiome may be limited in cutotype 2 compared with cutotype 1, which indicates the limited hydration functions of NMFs and explains the dry skin phenotype associated with cutotype 2 (Fig. 5). In addition to dryness, skin associated with cutotype 2 may be prone to other adverse conditions because taxa and KOs in cutotype 2 were also positively correlated with kynurenate, a key tryptophan derivative linked to pathological processes such as atopic dermatitis, which causes dry, itchy, and inflamed skin [48, 49]. Kynurenate could be derived from microbes to trigger host-microbe interactions as it is a ligand for the transcription factor aryl hydrocarbon receptor that regulates the functions of many skin cell types [50]. With increasing recognition of hydrocarbons in polluted air as an important factor in the development of atopic dermatitis, particularly in childhood [51–53], our results suggest that shifts in skin microbiome composition after air pollution exposure could contribute to the aggravation of skin dryness and inflammation.

While this study improved our understanding of the skin microbiome in the context of PAH exposure, it has some limitations. First, although the growth rates of microbial taxa can be inferred computationally, whether the taxa are alive or dead cannot be discerned from genomic DNA data alone. Second, there is little information on whether detected metabolites were derived from microbes, the host, or the environment. Third, multivariate analysis only reveals correlations but not causations such that observed associations may be ecologically significant or arise from stochastic processes [54]. Subsequently, the causal mechanisms underlying detected associations (e.g., adverse skin conditions) requires future experimental verification, such as by prospecting specific microbial–skin interplays using in vitro skin models colonized with microbial species from each cutotype and exposing them to different types and concentrations of PAHs [55–57]. Furthermore, multi-omics analyses including metatranscriptomics and metaproteomics together with metagenomics and metabolomics would provide finer understanding of the relationships between PAH exposure and microbial physiology, and the combination of culture-independent and culture-dependent assays would help to validate in silico associations [58].

## Conclusions

This study revealed the associations between PAH exposure and differentiation of skin microbiome into distinct cutotypes. Some of the differences in cutotypes may result in changes in microbial adaptation and skin–microbe interactions that affect host skin health. The coupling of PAH exposure and cutotype differentiation will help future studies to compartmentalize skin microbiomes into clusters. In-depth characterization of these clusters can then be performed to identify taxonomic, functional, or metabolic biomarkers associated with changes in skin phenotypes (e.g., acne onset, pigmentation frequency, and aging). Such a workflow will help develop strategies for preventing pollutant-associated skin effects by identifying specific features and interactions from microbiome clusters linked to pollutant exposure.

## Materials and methods

### Subject characteristics

The analyzed cheek samples originated from a cohort of female participants aged 25–45 years from the Chinese cities of Baoding (more severe air pollution) and Dalian (less severe) as part of a multi-objective study examining PAH accumulation in hair [59] and the associations of PAH exposure with signs of facial aging [36], skin microbial taxonomic composition [22], and metabolomes [11]. The participants resided in the respective cities for at least 15 years at the time of sampling. Details regarding cohort recruitment, inclusion and exclusion criteria, and sample collection procedures have been previously described [22]. For each subject, comprehensive skin physiological data collected through self-reporting and clinical assessment were also previously reported [22].

### Sequencing and quality control

Genomic DNA was extracted from sampling swabs using the PowerSoil DNA isolation kit (MO BIO Laboratories, Carlsbad, CA, USA) following the manufacturer’s instructions with minor modifications [22]. Library preparation and sequencing on an Ilumina NovaSeq platform to generate 150-bp paired-end reads were performed by SeqMatic LLC (Fremont, CA, USA) according to the manufacturer’s instructions. We processed and sequenced 124 samples (Baoding *n* = 61, Dalian *n* = 63) and four negative controls (i.e., new sterile swabs). The sequenced samples were selected across eight pollution exposure groups from weak to strong as previously described [11, 22] with 7 to 21 samples per group to obtain a balanced representation among exposure levels. Two samples from Baoding did not yield sufficient reads and were removed from downstream analysis. Sequence adapters were removed from the raw reads using AdapterRemoval (v2.3.1) [60] and quality filtering and human DNA read removal was performed using Kneaddata (v0.7.4) as previously described [22]. Sequencing quality information for the samples is shown in Supplementary Table 6. Following taxonomic classification of the reads using Kraken2 (v2.0.7-beta; kmer length = 35, confidence score threshold = 0) [61], then species-level abundance estimation using Bracken (v2.6.1; threshold for filter = 0) [62], putative contaminants were identified with the R package “decontam” based on the prevalence mode with 0.1 as the significance threshold [63]. This method deemed 65 species to be contaminants (Supplementary Table 6) and reads that could be mapped to the representative genomes of the contaminant species (Supplementary Table 6) were removed using an in-house script. Following quality filtering and contaminant removal, an average of 9,138,597 paired-end reads were retained per sample.

### Diversity and community composition analyses

Taxonomic classification of the filtered and contaminant-free reads was performed using Kraken2 and Bracken as described above. The species-level taxonomy was used to identify host factors associated with community diversity and compositional changes. Based on the taxonomic data, prediction strength analysis to estimate the optimal number of clusters in the community was performed based on the “partitioning around medoids” discrete clustering method [64] using the “prediction.strength” command (with 100 random splits) in the R package “fpc” (v2.2–9). The highest mean prediction strength was 0.81 when *k* = 2, where *k* is the number of clusters. This result formed the basis for the presence of two cutotypes among all samples. FindFungi (v0.23) [65] was used to assign taxonomy to the members of the fungal communities.

HUMAnN3 (v3.0.0.alpha.3) [66] was used to profile the potential metabolic functions of the metagenomes, revealing 507,517 UniRef90 gene families that were grouped into 361 MetaCyc functional pathways and 5564 KOs. Based on this UniRef90 pathway data, contributional diversity (Gini–Simpson for within-sample and Bray–Curtis for between-sample diversity) [28] was calculated for each cutotype and compared by selecting pathways detected in > 75% of samples within each cutotype. Antimicrobial resistance gene family markers were detected using ShortBRED (v0.9.3) [67]. The associations of the taxonomic, functional, and antimicrobial resistance data with host and environmental factors, including PAH exposure, were determined using MaAsLin2 [68] with the generalized linear model, with city, acne onset, wrinkle grade, facial pigmentation frequency, and exposure to PAHs as fixed effects and BMI and age as random effects. An adjusted *p* value (i.e., *q* value) ≤ 0.05 was considered statistically significant in this analysis. The composition differences (ß-diversity) between samples were analyzed based on the Bray–Curtis dissimilarity using the function “vegdist” in the R package “vegan,” and the permutational multivariate analysis of variance (PERMANOVA) test was applied using the “adonis” function in the R package “vegan” with 999 permutations to test the influence of factors including cutotype, city, acne onset, age group, and facial pigmentation frequency. The effects of PAHs on community differentiation by cutotype were assessed with distance-based redundancy analysis using the “capscale” function in the R package “vegan.” The Procrustes test was performed to determine congruency between taxonomic (Bracken) and functional (HUMAnN) composition data using the “protest” function in the R package “vegan.” To assess α-diversity, the samples were first subsampled using seqtk (v1.3) to a read depth of 313,504 reads per sample, which corresponded to the sequencing depth of the sample with the lowest number of reads. This depth was representative of the total species-level taxon richness of the metagenomes (Supplementary Fig. 3). Shannon diversity was calculated for the subsampled dataset in “vegan.” Statistical significance of comparisons between two and more than two groups were performed using the Mann–Whitney and Kruskal–Wallis tests, respectively, in the R package “stats” (v3.6.1). False discovery rate (FDR) adjustment of the statistical significance was performed using the Benjamini–Hochberg method.

### Community- and strain-level in situ growth rate estimation

Growth rate estimation of the community was performed using the Growth Rate Index (GRiD) algorithm [6] with a coverage cutoff (-c) of 0.2, and ambiguous reads were re-assigned using Pathoscope (v2.0) [69]. Growth rate estimation of subspecies was performed using the Strain-Level Metagenomic Estimation of Growth Rate (SMEG) (v1.1.5) algorithm [7]. Reference genomes for each species included in the SMEG analysis (Supplementary Table 7) were obtained from the RefSeq archive of NCBI (accessed 2 June 2020).

### Representation of metabolites in cutotypes

The representation of metabolites in each cutotype was characterized using a *v* test [11] with the “catdes” function in the “FactoMinerR” package. For continuous variables, we tested if the mean of a particular subgroup was different from the mean of the total population. For discrete or qualitative variables, we tested if the proportion of a modality in a particular subgroup was over-expressed or under-expressed compared with the total population.

### Correlation analysis of the multivariate datasets

For each cutotype, taxonomic (Bracken) and functional (HUMAnN) features of the microbiome as well as the measured skin metabolites and PAHs were incorporated into a multivariate analysis to identify the correlating features between the datasets. Residualized values of all features from each type of data were determined based on a linear mixed-effects model using the R package “lme4.” The model included city, acne onset, and facial pigmentation frequency as fixed effects and age group as a random effect. The resulting residualized datasets were subjected to pairwise hierarchical all-against-all association (HAllA) analysis (v0.8.18) as previously described [30]. Spearman’s rank correlations with a Benjamini–Hochberg FDR-corrected *p* value (i.e., *q* value) ≤ 0.25 were considered statistically significant [30]. Spearman’s correlations <|0.25| were considered weak and were removed from the output. A sub-network containing 500 correlations of the lowest *q* value within each HAllA analysis between two datasets was constructed for each cutotype. The sub-networks for the two cutotypes were merged and visualized in Cytoscape (v3.8.2) to identify both correlations shared between the cutotypes and cutotype-specific correlations [70].

### Measurement of PAHs in hair samples

The detailed methods for analyzing PAHs in hair samples to assess subjects’ pollutant exposure levels have been previously reported [59]. Briefly, hair samples were washed to remove any external deposits and then pulverized, hydrolyzed, extracted, and analyzed using gas and liquid chromatography coupled with tandem mass spectrometry (MS/MS). We quantified 15 PAHs (all part of the US-EPA priority list), nicotine, and cotinine.

### Measurement of skin metabolites

Detailed descriptions of the methods used for untargeted metabolomics analysis of skin samples have been previously described [11]. Briefly, the samples were extracted with methanol and then divided into four equal fractions. Two fractions were analyzed with separate reverse-phase (RP) ultra-performance liquid chromatography (UPLC)-MS/MS with positive ion mode electrospray ionization (ESI) optimized for hydrophilic and hydrophobic compounds, respectively. One fraction was analyzed with RP/UPLC-MS/MS with negative ion mode ESI, and one was analyzed with hydrophilic interaction liquid chromatography/UPLC-MS/MS with negative ion mode ESI. By comparing against a library of pure standards or using the analytical profiles (i.e., retention time, molecular weight, preferred adducts, and in-source fragments), 468 metabolites were identified. Those that could not be matched to known compounds were indicated with an X followed by a number.

## Supplementary Information

**Additional file 1: Table S1.** Factors to explain taxonomy, function, and resistome differences between skin microbiomes.

**Additional file 2: Table S2.** Enrichment of bacterial species-level taxa, growth rate, and functional and resistome features based on cutotype, host factors, and phenotypes.

**Additional file 3: Table S3.** Differences in the contributional diversity of core pathways between cutotypes.

**Additional file 4: Table S4.** Resistome of the skin microbiome grouped by sample and cutotype.

**Additional file 5: Table S5.** Properties of the multivariate correlation network by cutotype.

**Additional file 6: Table S6.** Quality control and contaminant removal information.

**Additional file 7: Table S7.** Reference genomes included for SMEG database construction.

**Additional file 8: Figure S1.** Bacterial and fungal microbiome of cheek samples by city. (a) Rank prevalence curve of the 1,525 species-level taxa detected in the 124 skin microbiome samples. The 42 species commonly detected in all samples (i.e., left edge of histogram) represent the core microbiome. (b) Shannon diversity based on the rarefied depth of 313,504 reads per sample between cities and organized by acne onset. Statistical significance of pairwise comparisons was based on the Mann–Whitney test. (c) Relative abundance of fungal taxa in the samples. Bars are colored by city. (d) Top 12 species-level fungal taxa according to relative abundance of the fungal community of each sample organized by city. Other fungal taxa were grouped into the “Minor/Unclassified” category. (e) PCoA plot based on the Bray–Curtis dissimilarity of microbiome composition. Each point represents a metagenomic sample colored by city.

**Additional file 9: Figure S2.** Over-representation of skin metabolites in the two cutotypes. The metabolites shown were significantly (*p* < 0.05) over-represented in cutotypes 1 and 2 according to the v-test. Metabolites are colored according to class.

**Additional file 10: Figure S3.** Rarefaction curves of sequencing reads after quality control. The plot shows the number of species identified in each sample as a function of sequencing depth. Samples were rarefied to an even sampling depth of 313,504 reads per sample (indicated by the vertical line) for α-diversity analysis.

## Data Availability

The raw paired-end metagenomics sequences have been deposited in NCBI’s BioProject under accession number PRJNA730653. Custom scripts and input and output files generated for analysis and figures as well as the reference genomes used for decontamination are available at GitHub page https://github.com/mhyleung/skin_microbiota_pah2. Requests for access to the datasets used for figures should be directed to PKHL.

## References

[1] Flowers L, Grice EA (2020). The skin microbiota: balancing risk and reward. Cell Host Microbe.

[2] Tomida S, Nguyen L, Chiu B-H, Liu J, Sodergren E, Weinstock GM, et al. Pan-genome and comparative genome analyses of *Propionibacterium acnes* reveal its genomic diversity in the healthy and diseased human skin microbiome. mBio. 2013;4(3):e00003–13. 10.1128/mBio.00003-13 PMC3663185 23631911

[3] Dréno B, Dagnelie MA, Khammari A, Corvec S (2020). The skin microbiome: a new actor in inflammatory acne. Am J Clin Dermatol.

[4] Paller AS, Kong HH, Seed P, Naik S, Scharschmidt TC, Gallo RL (2019). The microbiome in patients with atopic dermatitis. J Allergy Clin Immunol.

[5] Byrd AL, Deming C, Cassidy SKB, Harrison OJ, Ng W-I, Conlan S, et al. *Staphylococcus aureus* and *Staphylococcus epidermidis* strain diversity underlying pediatric atopic dermatitis. Sci Transl Med. 2017;9(397):eaal4651. 10.1126/scitranslmed.aal4651 PMC5706545 28679656

[6] Emiola A, Oh J (2018). High throughput in situ metagenomic measurement of bacterial replication at ultra-low sequencing coverage. Nat Commun.

[7] Emiola A, Zhou W, Oh J. Metagenomic growth rate inferences of strains in situ. Sci Adv. 2020;6(17):eaaz2299. 10.1126/sciadv.aaz2299 PMC7176420 32494636

[8] Oh J, Byrd AL, Park M, Kong HH, Segre JA (2016). Temporal stability of the human skin microbiome. Cell.

[9] Bouslimani A, Porto C, Rath CM, Wang M, Guo Y, Gonzalez A (2015). Molecular cartography of the human skin surface in 3D. Proc Natl Acad Sci U S A.

[10] Bouslimani A, da Silva R, Kosciolek T, Janssen S, Callewaert C, Amir A (2019). The impact of skin care products on skin chemistry and microbiome dynamics. BMC Biol.

[11] Misra N, Clavaud C, Guinot F, Bourokba N, Nouveau S, Mezzache S (2021). Multi-omics analysis to decipher the molecular link between chronic exposure to pollution and human skin dysfunction. Sci Rep.

[12] Li Z, Xia J, Jiang L, Tan Y, An Y, Zhu X (2021). Characterization of the human skin resistome and identification of two microbiota cutotypes. Microbiome.

[13] Alekseyenko AV, Perez-Perez GI, De Souza A, Strober B, Gao Z, Bihan M (2013). Community differentiation of the cutaneous microbiota in psoriasis. Microbiome.

[14] Patel AB, Shaikh S, Jain KR, Desai C, Madamwar D (2020). Polycyclic aromatic hydrocarbons: sources, toxicity, and remediation approaches. Front Microbiol.

[15] Araviiskaia E, Berardesca E, Bieber T, Gontijo G, Sanchez Viera M, Marrot L (2019). The impact of airborne pollution on skin. J Eur Acad Dermatol Venereol.

[16] Schikowski T, Hüls A (2020). Air pollution and skin aging. Curr Environ Health Rep.

[17] Lefebvre M-A, Pham D-M, Boussouira B, Qiu H, Ye C, Long X, et al. Consequences of urban pollution upon skin status. A controlled study in Shanghai area. Int J Cosmet Sci. 2016;38(3):217–23. 10.1111/ics.12270 26291783

[18] Lefebvre M-A, Pham D-M, Boussouira B, Bernard D, Camus C, Nguyen Q-L (2015). Evaluation of the impact of urban pollution on the quality of skin: a multicentre study in Mexico. Int J Cosmet Sci.

[19] Stec AA, Dickens KE, Salden M, Hewitt FE, Watts DP, Houldsworth PE (2018). Occupational exposure to polycyclic aromatic hydrocarbons and elevated cancer incidence in firefighters. Sci Rep.

[20] Sowada J, Schmalenberger A, Ebner I, Luch A, Tralau T (2014). Degradation of benzo[a]pyrene by bacterial isolates from human skin. FEMS Microbiol Ecol.

[21] Sowada J, Lemoine L, Schön K, Hutzler C, Luch A, Tralau T (2017). Toxification of polycyclic aromatic hydrocarbons by commensal bacteria from human skin. Arch Toxicol.

[22] Leung MHY, Tong X, Bastien P, Guinot F, Tenenhaus A, Appenzeller BMR (2020). Changes of the human skin microbiota upon chronic exposure to polycyclic aromatic hydrocarbon pollutants. Microbiome.

[23] Phale PS, Malhotra H, Shah BA (2020). Degradation strategies and associated regulatory mechanisms/features for aromatic compound metabolism in bacteria. Adv Appl Microbiol.

[24] Lu C, Hong Y, Liu J, Gao Y, Ma Z, Yang B (2019). A PAH-degrading bacterial community enriched with contaminated agricultural soil and its utility for microbial bioremediation. Environ Pollut.

[25] Leung MHY, Wilkins D, Lee PKH (2015). Insights into the pan-microbiome: skin microbial communities of Chinese individuals differ from other racial groups. Sci Rep.

[26] Ling Z, Liu X, Luo Y, Yuan L, Nelson KE, Wang Y (2013). Pyrosequencing analysis of the human microbiota of healthy Chinese undergraduates. BMC Genomics.

[27] Sun Y, Fu X, Li Y, Yuan Q, Ou Z, Lindgren T (2020). Shotgun metagenomics of dust microbiome from flight deck and cabin in civil aviation aircraft. Indoor Air.

[28] Franzosa EA, Mciver LJ, Rahnavard G, Thompson LR, Schirmer M, Weingart G (2018). Species-level functional profiling of metagenomes and metatranscriptomes. Nat Methods.

[29] Ro BI, Dawson TL (2005). The role of sebaceous gland activity and scalp microfloral metabolism in the etiology of seborrheic dermatitis and dandruff. J Investig Dermatol Symp Proc.

[30] Lloyd-Price J, Arze C, Ananthakrishnan AN, Schirmer M, Avila-Pacheco J, Poon TW (2019). Multi-omics of the gut microbial ecosystem in inflammatory bowel diseases. Nature.

[31] Roager HM, Licht TR (2018). Microbial tryptophan catabolites in health and disease. Nat Commun.

[32] Shibagaki N, Suda W, Clavaud C, Bastien P, Takayasu L, Iioka E (2017). Aging-related changes in the diversity of women’s skin microbiomes associated with oral bacteria. Sci Rep.

[33] Chien AL, Tsai J, Leung S, Mongodin EF, Nelson AM, Kang S (2019). Association of systemic antibiotic treatment of acne with skin microbiota characteristics. JAMA Dermatol.

[34] Prescott SL, Larcombe D-L, Logan AC, West C, Burks W, Caraballo L (2017). The skin microbiome: impact of modern environments on skin ecology, barrier integrity, and systemic immune programming. World Allergy Organ J.

[35] Huang N, Mi T, Xu S, Dadd T, Ye X, Chen G (2020). Traffic-derived air pollution compromises skin barrier function and stratum corneum redox status: a population study. J Cosmet Dermatol.

[36] Flament F, Bourokba N, Nouveau S, Li J, Charbonneau A. A severe chronic outdoor urban pollution alters some facial aging signs in Chinese women. A tale of two cities. Int J Cosmet Sci. 2018;40(5):467–81. 10.1111/ics.12487 30112861

[37] Patra V, Wagner K, Arulampalam V, Wolf P. Skin microbiome modulates the effect of ultraviolet radiation on cellular response and immune function. iScience. 2019;15:211–22. 10.1016/j.isci.2019.04.026 PMC6515114 31079025

[38] Arumugam M, Raes J, Pelletier E, Le Paslier D, Yamada T, Mende DR (2011). Enterotypes of the human gut microbiome. Nature.

[39] Costea PI, Hildebrand F, Arumugam M, Bäckhed F, Blaser MJ, Bushman FD (2018). Enterotypes in the landscape of gut microbial community composition. Nat Microbiol.

[40] Oh J, Byrd AL, Deming C, Conlan S, Program NCS, Kong HH (2014). Biogeography and individuality shape function in the human skin metagenome. Nature.

[41] Zhang G-Y, Ling J-Y, Sun H-B, Luo J, Fan Y-Y, Cui Z-J. Isolation and characterization of a newly isolated polycyclic aromatic hydrocarbons-degrading *Janibacter anophelis* strain JY11. J Hazard Mater. 2009;172(2–3):580–6. 10.1016/j.jhazmat.2009.07.037 19660861

[42] Sandhu M, Paul AT, Jha PN. Metagenomic analysis for taxonomic and functional potential of polyaromatic hydrocarbons (PAHs) and polychlorinated biphenyl (PCB) degrading bacterial communities in steel industrial soil. PLoS One. 2022;17(4): e0266808. 10.1371/journal.pone.0266808 PMC9053811 35486615

[43] Park T, Kim HJ, Myeong NR, Lee HG. Kwack I. Lee J, et al. Collapse of human scalp microbiome network in dandruff and seborrhoeic dermatitis. Exp Dermatol. 2017;26(9):835–8. 10.1111/exd.13293 28094891

[44] Kubo A, Ishizaki I, Kubo A, Kawasaki H, Nagao K, Ohashi Y (2013). The stratum corneum comprises three layers with distinct metal-ion barrier properties. Sci Rep.

[45] Sandilands A, Sutherland C, Irvine AD, Mclean WHI (2009). Filaggrin in the frontline: role in skin barrier function and disease. J Cell Sci.

[46] Liu X, Ge W, Zhang X, Chai C, Wu J, Xiang D, et al. Biodegradation of aged polycyclic aromatic hydrocarbons in agricultural soil by *Paracoccus* sp. LXC combined with humic acid and spent mushroom substrate. J Hazard Mater. 2019;379:120820. 10.1016/j.jhazmat.2019.120820 31271936

[47] Qi S-W, Chaudhry MT, Zhang Y, Meng B, Huang Y, Zhao KX, et al. Comparative proteomes of *Corynebacterium glutamicum* grown on aromatic compounds revealed novel proteins involved in aromatic degradation and a clear link between aromatic catabolism and gluconeogenesis via fructose-1,6-bisphosphatase. Proteomics. 2007;7(20):3775–87. 10.1002/pmic.200700481 17880007

[48] Guenin-Macé L, Morel J-D, Doisne J-M, Schiavo A, Boulet L, Mayau V (2020). Dysregulation of tryptophan catabolism at the host-skin microbiota interface in hidradenitis suppurativa. JCI Insight.

[49] Szelest M, Walczak K, Plech T (2021). A new insight into the potential role of tryptophan-derived AhR ligands in skin physiological and pathological processes. Int J Mol Sci.

[50] Van Den Bogaard EH, Esser C, Perdew GH (2021). The aryl hydrocarbon receptor at the forefront of host-microbe interactions in the skin: a perspective on current knowledge gaps and directions for future research and therapeutic applications. Exp Dermatol.

[51] Manisalidis I, Stavropoulou E, Stavropoulos A, Bezirtzoglou E (2020). Environmental and health impacts of air pollution: a review. Front Public Health.

[52] Ahn K (2014). The role of air pollutants in atopic dermatitis. J Allergy Clin Immunol.

[53] Wang C, Wei C-C, Wan L, Lin C-L, Tsai J-D (2021). Association of exposure to hydrocarbon air pollution with the incidence of atopic dermatitis in children. Ital J Pediatr.

[54] Faust K (2021). Open challenges for microbial network construction and analysis. ISME J.

[55] Larson PJ, Chong D, Fleming E, Oh J (2021). Challenges in developing a human model system for skin microbiome research. J Invest Dermatol.

[56] Niehues H, Bouwstra JA, El Ghalbzouri A, Brandner JM, Zeeuwen PLJM, Van Den Bogaard EH (2018). 3D skin models for 3R research: the potential of 3D reconstructed skin models to study skin barrier function. Exp Dermatol.

[57] Lemoine L, Bayrambey D, Roloff A, Hutzler C, Luch A, Tralau T. Commensal-related changes in the epidermal barrier function lead to alterations in the Benzo[a]Pyrene metabolite profile and its distribution in 3D skin. mBio. 2021;12(5):e01223–21. 10.1128/mBio.01223-21 PMC8546866 34579573

[58] Chng KR, Tay ASL, Li C, Ng AHQ, Wang J, Suri BK (2016). Whole metagenome profiling reveals skin microbiome-dependent susceptibility to atopic dermatitis flare. Nat Microbiol.

[59] Palazzi P, Mezzache S, Bourokba N, Hardy EM, Schritz A, Bastien P (2018). Exposure to polycyclic aromatic hydrocarbons in women living in the Chinese cities of BaoDing and Dalian revealed by hair analysis. Enviro Int.

[60] Schubert M, Lindgreen S, Orlando L (2016). AdapterRemoval v2: rapid adapter trimming, identification, and read merging. BMC Res Notes.

[61] Wood DE, Lu J, Langmead B (2019). Improved metagenomic analysis with Kraken 2. Genome Biol.

[62] Lu J, Breitwieser FP, Thielen P, Salzberg SL (2017). Bracken: estimating species abundance in metagenomics data. PeerJ Comput Sci.

[63] Davis NM, Proctor DM, Holmes SP, Relman DA, Callahan BJ (2018). Simple statistical identification and removal of contaminant sequences in marker-gene and metagenomics data. Microbiome.

[64] Kaufman L, Rousseeuw PJ. Partitioning around Medoids (Program PAM). In Finding groups in data: an introduction to cluster analysis. 1990. p. 68–125.

[65] Donovan PD, Gonzalez G, Higgins DG, Butler G, Ito K. Identification of fungi in shotgun metagenomics datasets. PLoS One. 2018;13(2):e0192898. 10.1371/journal.pone.0192898 PMC5812651 29444186

[66] Beghini F, Mciver LJ, Blanco-Míguez A, Dubois L, Asnicar F, Maharjan S, et al. Integrating taxonomic, functional, and strain-level profiling of diverse microbial communities with bioBakery 3. Elife. 2021;10:e65088. 10.7554/eLife.65088 PMC8096432 33944776

[67] Kaminski J, Gibson MK, Franzosa EA, Segata N, Dantas G, Huttenhower C (2015). High-specificity targeted functional profiling in microbial communities with ShortBRED. PLoS Comput Biol.

[68] Mallick H, Rahnavard A, Mciver LJ, Ma S, Zhang Y, Nguyen LH (2021). Multivariable association discovery in population-scale meta-omics studies. PLoS Comput Biol.

[69] Hong C, Manimaran S, Shen Y, Perez-Rogers JF, Byrd AL, Castro-Nallar E, et al. PathoScope 2.0: a complete computational framework for strain identification in environmental or clinical sequencing samples. Microbiome. 2014;2:33. 10.1186/2049-2618-2-33 PMC4164323 25225611

[70] Shannon P, Markiel A, Ozier O, Baliga NS, Wang JT, Ramage D (2003). Cytoscape: a software environment for integrated models of biomolecular interaction networks. Genome Res.

