# Supplementary material for: Skin microbiome differentiates into distinct cutotypes with unique metabolic functions upon exposure to polycyclic aromatic hydrocarbons
Source: Microbiome. 2023 Jun 1;11:124. doi: 10.1186/s40168-023-01564-4 (PMC10233911; doi:10.1186/s40168-023-01564-4)
Supplement: Supplementary file 9 — Additional file 8: Figure S1. Bacterial and fungal microbiome of cheek samples by city. (a) Rank prevalence curve of the 1,525 species-level taxa detected in the 124 skin microbiome samples. The 42 species commonly detected in all samples (i.e., left edge of histogram) represent the core microbiome. (b) Shannon diversity based on the rarefied depth of 313,504 reads per sample between cities and organized by acne onset. Statistical significance of pairwise comparisons was based on the Mann–Whitney test. (c) Relative abundance of fungal taxa in the samples. Bars are colored by city. (d) Top 12 species-level fungal taxa according to relative abundance of the fungal community of each sample organized by city. Other fungal taxa were grouped into the “Minor/Unclassified” category. (e) PCoA plot based on the Bray–Curtis dissimilarity of microbiome composition. Each point represents a metagenomic sample colored by city. [file 40168_2023_1564_MOESM8_ESM.docx]

**Supplementary Fig. 1. Bacterial and fungal microbiome of cheek samples by city. (a)** Rank prevalence curve of the 1,525 species-level taxa detected in the 124 skin microbiome samples. The 42 species commonly detected in all samples (i.e., left edge of histogram) represent the core microbiome. **(b)** Shannon diversity based on the rarefied depth of 313,504 reads per sample between cities and organized by acne onset. Statistical significance of pairwise comparisons was based on the Mann–Whitney test. **(c)** Relative abundance of fungal taxa in the samples. Bars are colored by city. **(d)** Top 12 species-level fungal taxa according to relative abundance of the fungal community of each sample organized by city. Other fungal taxa were grouped into the “Minor/Unclassified” category. **(e)** PCoA plot based on the Bray–Curtis dissimilarity of microbiome composition. Each point represents a metagenomic sample colored by city.
