# Supplementary material for: Skin microbiome differentiates into distinct cutotypes with unique metabolic functions upon exposure to polycyclic aromatic hydrocarbons
Source: Microbiome. 2023 Jun 1;11:124. doi: 10.1186/s40168-023-01564-4 (PMC10233911; doi:10.1186/s40168-023-01564-4)
Supplement: Supplementary file 10 — Additional file 9: Figure S2. Over-representation of skin metabolites in the two cutotypes. The metabolites shown were significantly (p < 0.05) over-represented in cutotypes 1 and 2 according to the v-test. Metabolites are colored according to class. [file 40168_2023_1564_MOESM9_ESM.docx]

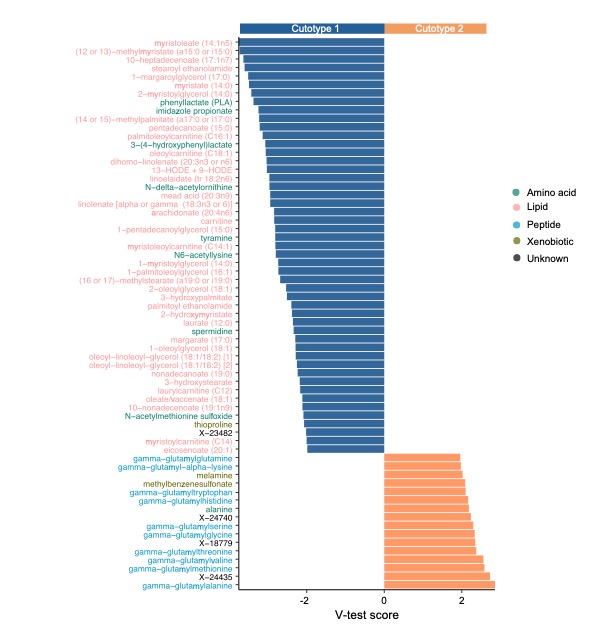


**Supplementary Fig. 2. Over-representation of skin metabolites in the two cutotypes.** The metabolites shown were significantly (*p* $<$0.05) over-represented in cutotypes 1 and 2 according to the v-test. Metabolites are colored according to class.
