# Supplementary material for: Skin microbiome differentiates into distinct cutotypes with unique metabolic functions upon exposure to polycyclic aromatic hydrocarbons
Source: Microbiome. 2023 Jun 1;11:124. doi: 10.1186/s40168-023-01564-4 (PMC10233911; doi:10.1186/s40168-023-01564-4)
Supplement: Supplementary file 11 — Additional file 10: Figure S3. Rarefaction curves of sequencing reads after quality control. The plot shows the number of species identified in each sample as a function of sequencing depth. Samples were rarefied to an even sampling depth of 313,504 reads per sample (indicated by the vertical line) for α-diversity analysis. [file 40168_2023_1564_MOESM10_ESM.docx]

**
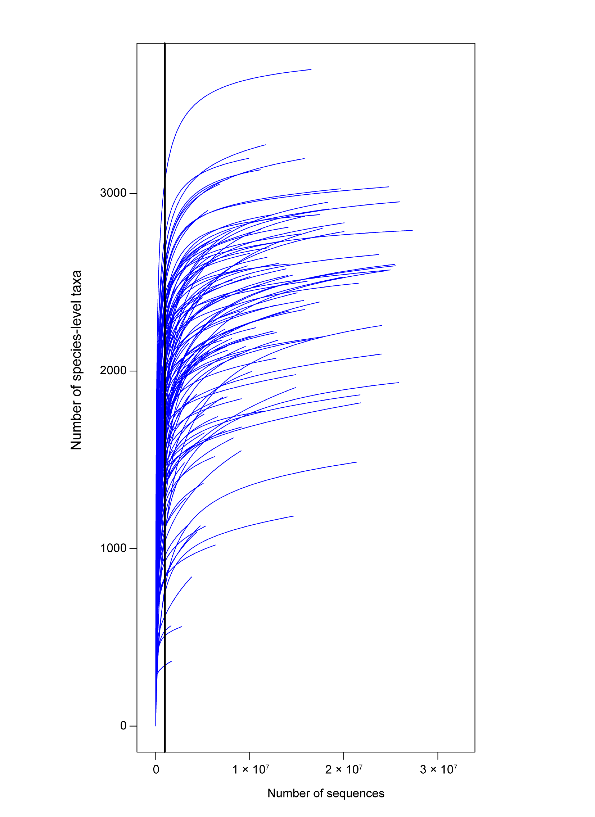
**

**Supplementary Fig. 3. Rarefaction curves of sequencing reads after quality control.** The plot shows the number of species identified in each sample as a function of sequencing depth. Samples were rarefied to an even sampling depth of 313,504 reads per sample (indicated by the vertical line) for α-diversity analysis.
